# Euthanasia, religiosity and the valuation of health states: results from an Irish EQ5D5L valuation study and their implications for anchor values

**DOI:** 10.1186/s12955-018-0985-9

**Published:** 2018-07-31

**Authors:** Luke Barry, Anna Hobbins, Daniel Kelleher, Koonal Shah, Nancy Devlin, Juan Manuel Ramos Goni, Ciaran O’Neill

**Affiliations:** 1 J.E. Cairnes School of Business and Economics, NUI, Galway, Ireland; 2 Center for Public Health, Queens University Belfast, Belfast, BT12 6BA Northern Ireland; 3 Office of Health Economics, London, England; 4 EuroQol Research Foundation, Rotterdam, Holland

**Keywords:** Euthanasia, Religion, EQ5D5L, Anchor states, Worse than dead, Ireland

## Abstract

**Background:**

The Quality Adjusted Life Year influences the allocation of significant amounts of healthcare resources. Despite this surprisingly little research effort has been devoted to analysing how beliefs and attitudes to hastening death influence preferences for health states anchored at “dead” and “perfect health”. In this paper we examine how, inter alia, adherence to particular religious beliefs (religiosity) influences attitudes to euthanasia and how, inter alia, attitudes to euthanasia influences the willingness to assign worse than dead (WTD) values to health states using data collected as part of the Irish EQ5D5L valuation study.

**Methods:**

A sample of 160 respondents each supplied 10 composite time trade-off valuations and information on religiosity and attitudes to euthanasia as part of a larger national survey. Data were analysed using a recursive bivariate probit model in which attitudes to euthanasia and willingness to assign WTD values were analysed jointly as functions of a range of covariates.

**Results:**

Religiosity was a significant determinant of attitudes to euthanasia and attitudes to euthanasia were a significant determinant of the likelihood of assigning WTD values. A significant negative correlation in errors between the two probit models was observed indicative of support for the hypothesis of endogeneity between attitudes to euthanasia and readiness to assign WTD values.

**Conclusion:**

In Ireland attitudes and beliefs play an important role in understanding health state preferences. Beyond Ireland this may have implications for: the construction of representative samples; understanding the values accorded health states and; the frequency with which value sets must be updated.

**Electronic supplementary material:**

The online version of this article (10.1186/s12955-018-0985-9) contains supplementary material, which is available to authorized users.

“Even death is unreliable, instead of zero it may be some ghastly hallucination such as the square root of minus one” Samuel Beckett

## Background

Quality adjusted life years (QALYs) have become an integral part of resource allocation decisions in many healthcare systems [1, 2]. In principle they provide a measure of health incorporating both longevity and utility that can be related to costs in the comparative analysis of alternative uses of healthcare resources [3]. The elicitation of preferences for use in QALYs generally involves the Time Trade-Off (TTO) technique. In these exercises, the utility associated with health states anchored at “dead” (with a value of 0) and perfect health (with a value of 1) [4–6] are elicited from members of the public and representative national value sets based on elicited values produced [7–9]. While the TTO approach has been in use for over 30 years [10], debate continues around the use of “dead” as an anchor value in it [11, 12]. What it means to “be” dead - and by extension how a given health state can be compared relative to this – it is argued, presents conceptual challenges regarding its use as an anchor state [11]. If being dead, for example, involves non-existence this implies the comparison of a health state with a non-state which is conceptually challenging. To extend Barrie’s analogy comparing health states with *being* dead is more akin to comparing apples with the square root of minus one than to comparing apples and oranges. Clearly though beliefs about *being* dead and attitudes to ending life are pertinent to these considerations.

While such arguments are interesting, in reality individuals appear willing to make such comparisons and indeed, it could be argued, act on the basis of them. The notion of rational suicide, for example, argues that some individuals who take their own lives do so based on a rational assessment that being dead is preferable to enduring their current state of existence - or what that state may become [13–16]. Indeed the legal protection afforded those who assist others in ending their lives in seven states of the USA (Washington, Oregon, Colorado, Vermont, Montana, Washington DC and California) as well as Canada, Luxembourg, the Netherlands, Germany, and Switzerland might be considered to be an implicit recognition of the possibility of a life that is less preferable to being dead and that individuals are capable of rational informed choices based on a comparison of these choices. In Ireland currently euthanasia and assisted suicide are illegal [17]. While suicide itself is no longer a criminal act since the passing of the Criminal Law (Suicide) Act of 1993, assisting in the suicide of anothercarries a sentence of up to fourteen years in prison where an indictment of murder or manslaughter can be brought against a person who may have ‘aided, abetted, counselled or procured the suicide of the person alleged to have been killed’ [18].

The subject of hastening death whether by suicide or euthanasia is emotive, complex and contentious but it is related to the thought experiment contained within TTO exercises in which death is hypothetically hastened through time trades. Given this, it is perhaps surprising that the role of beliefs, religiosity and attitudes to issues such as suicide – positions one might expect to inform perceptions of what being dead means or how it might be valued relative to other states - have not featured more prominently in an examination of how individuals value health states within TTO elicitation exercises. Just three studies that we are aware have sought to explicitly model the role of beliefs regarding an afterlife, religiosity and/or attitudes to actions that hasten death such as euthanasia, in TTO valuations [19–21].

In the study by Van Nooten et al. [19] the amount of time individuals in the Netherlands traded in TTO exercises was found to be higher among those who favoured access to euthanasia than those who opposed access to it under any circumstances. One might infer from this that individuals who favour access to euthanasia are more likely to accept the possibility of health states that are “worse than being dead” and trade more time including time they don’t have (i.e. they would rather be dead now) than experience those states. Similar results were found by Augestad et al. [21] in Norway, where those who favoured access to euthanasia traded more time than those who opposed access to euthanasia; here attitudes to euthanasia were measured on a scale rather than dichotomously as in van Nooten [19].

With respect to beliefs, in the Netherlands Van Nooten et al. [19] found no relationship between beliefs in an afterlife and the amount of time traded by respondents. By contrast Jakubczyk et al., [20] examining preferences in Poland found that those with a belief in the afterlife – the vast majority of whom the authors considered to be Roman Catholic - were less likely to trade time than those without such a belief. The contrasting results are interesting. The lack of significance among the Dutch could be explained by greater heterogeneity in religious beliefs which may in turn be related either to heterogeneity in the vehemence with which particular faiths oppose actions that hasten death and/or the degree of attachment (religiosity) to those faiths amongst the respondents. While 96% of the population in Poland is identified as Roman Catholic [22] for example, in the Netherlands just 31% are Roman Catholic and approximately 40% have no religious affiliation [23] suggesting greater heterogeneity of beliefs. Similarly, differences in the degree of religiosity (and thus how strongly religious denomination may be indicative of actual beliefs) are evident between the two countries. While approximately 57% of Poles actively participate in religious ceremonies at least once per week [22] in the Netherlands approximately 70% of the population never attend religious services [23]. Clearly this suggests that the context in which relationships between beliefs/attitudes and time traded are examined is important in understanding observed relationships. While not explicitly examining trades a study by Elbarazi et al. [24] also found in a study of 119 Muslims who completed a TTO survey in the United Arab Emirates that 81% reported their responses were somewhat or heavily influenced by their spiritual or religious beliefs supporting the contention that beliefs may have a role.

That van Nooten’s results in respect of beliefs contrast with those on euthanasia is also interesting. They suggest that while religious affiliation and attitudes to euthanasia may be correlated, they are distinct and may have distinct effects on time traded [19]. Evidence points to a relationship between religiosity and attitudes to euthanasia/assisted suicide [25, 26] but that individuals, even a majority of them, can hold opinions that diverge from the teaching of the faith they self-identify with is also clear [27]. This suggests that while indicators of beliefs (such as religion) or the strength of those beliefs (such as religiosity) can provide information pertinent to the perception of death and the willingness to trade time for health states around it, the strength of the relationship with the underlying belief/attitude of interest can vary and similar positions grounded other than in religious beliefs can be held. It thus becomes important to examine attitudes and beliefs as well as the vehemence with which these are held mindful of the potential interaction between them.

How anchor states are understood clearly has implications for how health states are valued relative to them and by extension how the quality part of QALYs are determined and resources subsequently allocated [28]. In this paper we examine the relationship between the likelihood of trading below zero – that is assigning a “worse than dead” (WTD) value to a health state – and a range of covariates including the religiosity and attitudes to euthanasia of the respondent. We discuss the implications of our results for the generation of value sets and the frequency with which these are revised.

## Methods

### Data

As part of a national survey of health preferences conducted using the EuroQol valuation technology [29] 160 respondents completed an additional survey that provided details on religion, attitudes to euthanasia and religiosity. Individuals were recruited to the study from a stratified random sample of small areas with random recruitment of households and individuals within households in each area. Further details of the study design, sample selection and quality assurance procedures are reported in Hobbins et al., [30]. Respondents were interviewed in person using a computer assisted personal interview process and completed 10 TTO valuations for randomly selected health states from among blocks described using the EQ5D5L descriptive system. In this, health is described at five levels (no problems, slight problems, moderate problems, severe problems, inability/extreme problems) across five domains (mobility, self-care, usual activities, pain/discomfort, anxiety/depression). A composite TTO exercise was used to elicit preferences for health states described using this system which is a combination of conventional TTO for health states that are considered better than dead and lead-time TTO for health states considered worse than dead by the participant [31].

Attitudes to euthanasia were elicited using the question: “in the case of a painful incurable disease should a doctor be allowed to end a patient’s life if they request it”. Responses were coded as 1 if yes and 0 if no with those responding ‘don’t know’ (of whom there were 18) being dropped from the analysis. Analyses were repeated with “don’t knows” recoded as being opposed to euthanasia in sensitivity analyses. Religiosity was established with a question “apart from special occasions, such as weddings or funerals, how often nowadays do you attend services or meetings connected with your religion”. Responses were represented in three dummy variables; less than at least monthly; at least monthly but less than at least weekly; and at least weekly. In each instance the dummy took the value 1 if yes and 0 otherwise.

Respondents also supplied details of their age, gender, marital status, education, current health status, whether they had dependents aged under 18, whether or not they had ever experienced a serious illness and which religious faith (if any) they identified themselves as belonging to. Age was coded as a series of groups: 18–35; 36–45; 46–60 and 61 plus, based on roughly equal distributions and coded as dummy variables equal to 1 if a member of the group and 0 otherwise. Gender was coded as 1 if male and 0 if female. The highest level of educational attainment was coded as a dummy variable equal to 1 if third level education was attained and 0 otherwise. Marital status was defined as married or living as married and coded a 1 if yes and 0 otherwise. Respondent’s health was represented using a visual analogue scale with values ranging from zero to 100. The respondent was also asked if they themselves had ever experienced a serious illness, which was coded as 1 if yes and 0 if no. Whether the respondent had dependents aged under 18 was code as 1 if yes and 0 otherwise. As 95% of the sample who identified their religion self-identified as Roman Catholic the role of religious denomination was not examined.

### Data analysis

Time traded was examined with reference to the probability of the respondent assigning values equivalent to worse than dead – values less than zero in the TTO. To allow for possible endogeneity between attitudinal responses a recursive bivariate probit model was developed [32]. Support for euthanasia was specified as a function of age, gender, marital status, whether the respondent had dependents aged under 18, education, visual analogue score for self-reported health, personal experience of a serious illness and religiosity. Willingness to assign a WTD value to a health state (10 per individual) was given the value 1 if yes and 0 otherwise and specified as a function of education, age, gender, marital status, the health state being valued, self-reported health (as measured on a visual analogue scale), personal experience of a serious illness whether the respondent had dependents aged under 18 and support for euthanasia. To account for multiple observations on the same individual, the analysis clustered on individual fixed effects. In sensitivity analyses models were re-estimated without clustering of standard errors. The health state valued was specified as a series of dummy variables based on the EQ5D5L framework. In this, the five domains of health – mobility, self-care, usual activities, pain/discomfort, and anxiety/depression – are represented at five levels of severity of impairment. A dummy variable was specified for each level with the highest level of health representing the base category in the regression.

The model assumes that the random components of the two regression equations are correlated, that is, that there are unobservable factors that influence the individual’s attitudes to euthanasia and assigning a WTD value. The potential endogeneity of attitudes to euthanasia and assignment of WTD values in the recursive model is reflected in the correlation in the error terms. We excluded religiosity from our WTD model while including it in our euthanasia model based on the assumption that religious teaching and thus the role of religiosity is likely to be more directly evident with respect to euthanasia than the TTO thought experiment. In sensitivity analyses models were re-estimated with equal to dead valuations (0’s) being treated as WTD. For comparative purposes a reduced form probit model in which WTD was specified as function of the covariates already identified with religiosity replacing attitudes to euthanasia was also estimated.

## Results

In Table 1 descriptive statistics for the sample are presented. As can be seen while over 40% of the sample attended religious services on at least a monthly basis, 75% favoured access to physician provided euthanasia for someone with a painful incurable disease who requested it.

**Table 1:** Descriptive Statistics

Sociodemographic Variables	
Religiosity (How often do you attend services)	
A few times a year or less	57%
At least once a month	14%
At least once a week	28%
Age Group (yrs)	
18–35	28%
36–45	28%
46–60	21%
61+	24%
Third Level Education	61%
Male	36%
Married/Living as Married	62%
Dependants Under 18 years	46%
In favour of Legalisation of Euthanasia (see scenario in Fig. 1)	75%
Experienced a serious illness	27%
Visual Analogue Scale (mean)	81.47
	*N* = 160

**Fig. 1:**
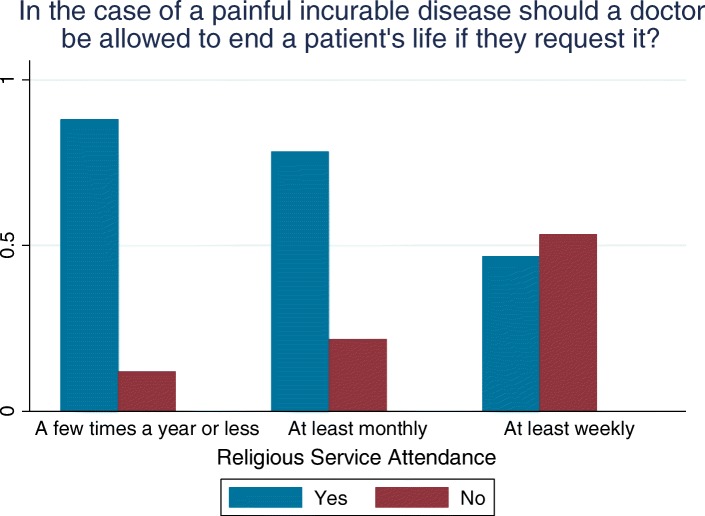
Proportion of those in favour or against euthanasia (see scenario below) across religiosity

In Fig. 1 the correlation in attitudes to euthanasia and religiosity is clearly evident – opposition increasing with frequency of religious service attendance. Interestingly though even among those who attend services at least weekly a significant proportion – over 40% - favoured access to euthanasia under the circumstances described.

In Table 2 the results of the regression analysis are presented. As can be seen from the significance of rho there is a strong negative correlation in residuals between the two regressions. This clearly indicates a linkage between attitudes in the two functions.

**Table 2:** Regression results for recursive bivariate probit models

Dependant Variable	Worse-than-Dead		Euthanasia	
	Coefficient	Standard Error	Coefficient	Standard Error
Third Level Education	−0.155	(0.167)	−0.077	(0.255)
Age Group (Base: 18–35)				
36–45	0.304	(0.207)	0.328	(0.32)
46–60	0.471*	(0.228)	0.318	(0.42)
61+	1.168***	(0.274)	0.585	(0.489)
Male	−0.298	(0.17)	−0.113	(0.251)
Married/Living as Married	−0.158	(0.204)	−0.07	(0.303)
Dependants Under 18 (Y/N)	−0.098	(0.208)	0.412	(0.359)
Visual Analogue Scale	0.004	(0.006)	−0.012	(0.008)
Experienced a serious illness	−0.287	(0.264)	−0.765*	(0.3)
In favour of Legalisation of Euthanasia (see scenario in Fig. 1)	1.003*	(0.394)	–	–
Religiosity (Base: A few times a year or less)				
Monthly	–	–	−0.454	(0.318)
Weekly	–	–	−1.369***	(0.328)
Mobility (Base: No Problems)				
Slight problems	0.194	(0.114)	–	–
Moderate problems	0.326**	(0.109)	–	–
Severe problems	0.476***	(0.12)	–	–
Unable	0.419***	(0.112)	–	–
Self-care (Base: No Problems)				
Slight problems	0.263*	(0.128)	–	–
Moderate problems	0.34*	(0.131)	–	–
Severe problems	0.528***	(0.143)	–	–
Unable	0.444***	(0.102)	–	–
Usual Activities (Base: No Problems)				
Slight problems	0.304*	(0.134)	–	–
Moderate problems	0.477**	(0.156)	–	–
Severe problems	0.443***	(0.118)	–	–
Unable	0.335***	(0.091)	–	–
Paid/Discomfort (Base: No Problems)				
Slight problems	0.253*	(0.116)	–	–
Moderate problems	0.182	(0.116)	–	–
Severe problems	0.614***	(0.111)	–	–
Extreme problems	0.852***	(0.138)	–	–
Anxiety/Depression (Base: No Problems)				
Slight problems	0.221	(0.128)	–	–
Moderate problems	0.313*	(0.14)	–	–
Severe problems	0.867***	(0.159)	–	–
Extreme problems	0.858***	(0.133)	–	–
Constant	−3.331***	(0.626)	2.05**	(0.748)
Rho^a^			−0.801*	(0.402)
Number of observations^a^				1600
Number of clusters^a^				160

The table shows the relationship between the dependent and independent variables in a recursive bivariate probit regression. Coefficients can be interpreted in the usual manner – the significant positive coefficient associated with being aged 61+ for example shows that the probability of assigning a WTD value is higher among this age group relative to those aged 18–35. Similarly, the negative and significant coefficient on those who attend religious services weekly compared to those who attend a few times a year or less, shows that the frequently attending group are less likely to support access to euthanasia than the infrequently attending group. Other coefficients can be interpreted in a similar fashion. The negative and significant value for Rho shows the correlation in errors between the two regressions is negative and large (0.8 in absolute terms)

^a^Pertains to both models

*p < 0.05, **p < 0.01, ***p < 0.001

With respect to other results these are broadly consistent with intuition, the likelihood of assigning a WTD value, for example, increasing with the severity of the health state posited. The significance of religiosity in attitudes to euthanasia is evident. So too is the significance of attitudes to euthanasia in explaining the probability of the respondent being willing to assign a WTD value to a health state. The model in which equivalent to dead (ETD) is treated as WTD (Additional file 1: Table S1) and the probit model of WTD valuations as a function of covariates shown with religiosity replacing euthanasia as a covariate are presented in the supplementary material (Additional file 1: Table S2). Sensitivity analyses in which standard errors were not clustered to account for multiple observations on the same person resulted in smaller standard errors as expected. These results are not re-produced in the interests of brevity but are available on request from the authors.

## Discussion

TTO is used to obtain health state values that reflect the preferences of respondent’s for particular states relative to anchor states. Attitudes and beliefs regarding those anchor states and in particular the readiness to consider a health state as being worse than dead may have a bearing on the relative value assigned all health states within the TTO framework. The results reported in Table 2 clearly indicate the utility of incorporating attitudes to euthanasia and strength of affiliation to a set of beliefs (measured here as religiosity) in understanding the propensity to assign worse than dead values. These results remained robust where ETD was treated as WTD (Additional file 1: Table S1). As with van Nooten [19], it is important to remember that this result though is context specific, that is, whether this would be similarly the case in more religiously diverse or secular countries or where the circumstances under which it is considered legitimate to hasten death differed is unclear. Almost 90% of those resident in Ireland self-identify as Roman Catholics [33] as did approximately 95% of our sample. While the possibility of a relationship between faith and values has been noted in other contexts [20, 24] there is evident heterogeneity in the nature of those relationships. Similarly, while the circumstances under which death might be hastened in this context referred to a painful and incurable disease, in other contexts other circumstances might operate and affect decisions differently.

Within the Irish context that both religiosity and attitudes to euthanasia help to explain the propensity to assign WTD values is evident and is consistent with intuition. Those evidencing greater religiosity, one might reasonably assume within the context of a predominantly Roman Catholic sample, are more likely to accept religious teaching that life has an intrinsic value regardless of the condition in which it is experienced. It seems reasonable to infer that such individuals are less likely to support access to services that would hasten death where the quality of that life is low and/or its duration likely to be short, or to assign values to a health state that equate it with being worse than dead (Additional file 1: Table S2), though religiosity is seen to be less significant and the magnitude of its effect is lower compared to euthanasia in Table 2. Whether religiosity determines attitudes to euthanasia or attitudes to issues such as euthanasia determines religiosity (those who hold certain beliefs attend religious services more frequently or those who attend religious services more frequently are more likely to hold certain beliefs) is unclear. In practice it is probable that directionality works in both ways and is mutable as attitudes change and beliefs are challenged by life experiences [34, 35].

The importance of examining attitudes and beliefs jointly though is evident from the analysis and the significance of rho in the bivariate probit. Its negative sign suggests that where we over-predict support for euthanasia we under-predict willingness to assign WTD values. This result can be interpreted with reference to unobserved variables omitted from the analysis. For example, it is conceivable that religiosity and attitudes to euthanasia capture incompletely various factors pertinent to their relationship with traded times. What might be called egotism, conceit or lack of empathy on the part of the respondent, is unobserved but could be related to religiosity and attitudes to issues such as euthanasia. For example, a readiness to discount the value of the life of another in a compromised health state while seeing one’s own life as having value regardless of the state in which it is experienced could see one more likely to support euthanasia and less likely to assign a WTD value. If so, we might under-predict the egotist’s support for euthanasia and over-predict their readiness to assign WTD values in respect of their own health. Equally and equally plausibly, it could be that concerns for autonomy could be unobserved and related to religiosity and attitudes to euthanasia. A failure to properly control for support for autonomy could see us under-predict support for euthanasia while simultaneously over-predicting the readiness of respondents to assign WTD values. These explanations are of course speculative; our inability to be more definitive as to the reasoning behind the unobserved heterogeneity representing a limitation of our analysis. Nevertheless our study serves to illustrate the range of factors that might affect the assignment of WTD values and the challenges in modelling these with relatively blunt instruments such as religiosity and attitudes to issues such as euthanasia.

As well as providing insight into the assignment of WTD values in TTO experiments our results may have broader implications for the generation of nationally representative value sets. The production of a representative value set for use with instruments such as the EQ5D5L assumes that a representative sample can be recruited or estimates from a large sample generated that reflect the values of the society they will be used with. While samples can be shown to reflect the society from which they are drawn with respect to observable traits – age, gender, socio-economic status for example – less observable but equally important traits such as religious/moral/ethical beliefs, the importance attached to autonomy, social cohesion, experience with death and expectations of life among many factors - probably inform decisions around stated preferences in TTO and discrete choice experiments. It would be impossible to demonstrate that a sample was representative with respect to all of these attributes. The best protection is perhaps to hope such variables correlate with others that can be observed, upon which selection can take place and representativeness demonstrated.

As beliefs and mores change over time so too will the values that inform preferences and the readiness to trade time including the assignment of WTD values. Given this it is important that value sets such as those that underpin the EQ5D5L are updated regularly. Immigration, secularisation, population aging, economic growth (and decline), or a society’s coming to terms with previously denied histories could make value sets collected at one time a poor reflection of current values. Given the amount of healthcare resources whose allocation are informed by such sets [1] though it is clearly important that these updates occur.

### Limitations

Our study has a number of limitations that should be borne in mind. First, it may not be possible to generalise from our study as to the role of religiosity, attitudes and beliefs to other contexts. While Ireland has undergone immense change in the last 20 years economically, culturally and socially for a variety of reasons it may still differ in terms of the religious homogeneity and role of religiosity compared even to other European countries. This similarly applies with respect to the precise meaning of the term euthanasia, which can vary between contexts as by inference might attitudes to it when assigning WTD values.

Second, while data were collected using an internationally recognised protocol with external quality control provided by the EuroQol Research Foundation our analysis is based on just 160 individuals on whom we had both TTO and attitudinal data. This is a relatively small sample size though it still yielded significant results.

Third, we control for a limited number of attitudinal variables in our analysis. We don’t control, for example, for experience of the death of loved ones or of having cared for individuals approaching the end of their life. Such factors as well as personality traits such as empathy and egotism may, as we speculate, have important roles in shaping values and in trading time. While this is conceded, inevitably some variables will be omitted from any analysis and measurement issues will pertain to those that are included. With 160 observations and within the constraints imposed by respondent fatigue (the elicitation process typically took 45 min to complete) the analysis presented, we contend continues to provide valuable insights.

Finally, willingness to trade time to avoid health problems, while consistent with the QALY framework, is simply the mechanism used to estimate utility, rather than the end in itself. A respondent may be unwilling to give WTD values (for religious or other reasons) yet may otherwise have similar views regarding the undesirability of a given health state to another respondent who is less averse to trading time and giving WTD values. This is a limitation of the TTO method and one we are compelled to work within.

## Conclusions

We have shown that attitudes and beliefs can have a significant role in the determination of anchor states in the EQ5D5L. As the allocation of significant amounts of healthcare resources are informed by the valuation of health states relative to these anchors it is important that we understand their role. As societies and the values of those societies’ change, so too may positions with respect to the anchor state “dead” and with it the relevance of value sets generated in a country’s past. Similarly, where differences exist between jurisdictions with respect to such beliefs/attitudes care is warranted in transferring values sets between contexts. Despite the importance of QALYs in resource allocation, the role of beliefs/attitudes in the value sets underpinning them remains a neglected area of research.

## Additional file

Additional file 1: **Table S1.** Regression results for bivariate probit models with ‘Equal to Dead’ grouped with ‘Worse than Dead’. **Table S2.** Regression results for probit model with attitudes to euthanasia substituted for religiosity. (DOCX 23 kb)

## Abbreviations

EQ5D5L: EuroQol 5 Domains 5 Levels
ETD: Equivalent to Dead
QALY: Quality adjusted life years
TTO: Time Trade-Off
USA: United States of America
WTD: Worse than Dead
