# Supplementary material for: Euthanasia, religiosity and the valuation of health states: results from an Irish EQ5D5L valuation study and their implications for anchor values
Source: Health Qual Life Outcomes. 2018 Jul 31;16:152. doi: 10.1186/s12955-018-0985-9 (PMC6069795; doi:10.1186/s12955-018-0985-9)
Supplement: Supplementary file 1 — Table S1. Regression results for bivariate probit models with ‘Equal to Dead’ grouped with ‘Worse than Dead’. Table S2. Regression results for probit model with attitudes to euthanasia substituted for religiosity. (DOCX 23 kb) [file 12955_2018_985_MOESM1_ESM.docx]

**Table S1** Regression results for bivariate probit models with ‘Equal to Dead’ grouped with ‘Worse than Dead’

| Dependant Variable | WTD |  | Euthanasia |  |
| --- | --- | --- | --- | --- |
|  | Coefficient | Standard Error | Coefficient | Standard Error |
| Third Level Education | -0.147 | (0.167) | -0.075 | (0.253) |
| Age Group (Base: 18-35) |  |  |  |  |
| 36-45 | 0.297 | (0.21) | 0.325 | (0.32) |
| 46-60 | 0.409 | (0.232) | 0.316 | (0.419) |
| 61+ | 1.157*** | (0.279) | 0.582 | (0.488) |
| Male | -0.32 | (0.17) | -0.121 | (0.249) |
| Married/Living as Married | -0.118 | (0.199) | -0.069 | (0.301) |
| Dependants Under 18 (Y/N) | -0.134 | (0.211) | 0.409 | (0.357) |
| VAS | 0.005 | (0.006) | -0.012 | (0.008) |
| Experienced a serious illness | -0.242 | (0.249) | -0.778** | (0.297) |
| In favour of Legalisation of Euthanasia (see scenario in Fig. 1) | 1.088** | (0.343) | - | - |
| Religiosity (Base: A few times a year or less) |  |  |  |  |
| Monthly | - | - | -0.473 | (0.309) |
| Weekly | - | - | -1.368*** | (0.324) |
| Mobility (Base: No Problems) |  |  |  |  |
| Slight problems | 0.193 | (0.114) | - | - |
| Moderate problems | 0.305** | (0.107) | - | - |
| Severe problems | 0.453*** | (0.118) | - | - |
| Unable | 0.428*** | (0.116) | - | - |
| Self-care (Base: No Problems) |  |  |  |  |
| Slight problems | 0.296* | (0.124) | - | - |
| Moderate problems | 0.372** | (0.131) | - | - |
| Severe problems | 0.515*** | (0.14) | - | - |
| Unable | 0.478*** | (0.1) | - | - |
| Usual Activities (Base: No Problems) |  |  |  |  |
| Slight problems | 0.37** | (0.132) | - | - |
| Moderate problems | 0.514** | (0.156) | - | - |
| Severe problems | 0.491*** | (0.119) | - | - |
| Unable | 0.35*** | (0.09) | - | - |
| Paid/Discomfort (Base: No Problems) |  |  |  |  |
| Slight problems | 0.261* | (0.116) | - | - |
| Moderate problems | 0.165 | (0.111) | - | - |
| Severe problems | 0.6*** | (0.109) | - | - |
| Extreme problems | 0.88*** | (0.135) | - | - |
| Anxiety/Depression (Base: No Problems) |  |  |  |  |
| Slight problems | 0.291* | (0.126) | - | - |
| Moderate problems | 0.33* | (0.14) | - | - |
| Severe problems | 0.899*** | (0.164) | - | - |
| Extreme problems | 0.903*** | (0.138) | - | - |
| Constant | -3.525*** | (0.574) | 2.099** | (0.744) |
|  |  |  |  |  |
| Rho^ |  |  | -0.923* | (0.397) |
| Number of observations^ |  |  |  | 1600 |
| Number of clusters^ |  |  |  | 160 |
| ^ Pertains to both models |  |  |  |  |

*p < 0.05, **p < 0.01, ***p < 0.001

**Table S2** Regression results for probit model with attitudes to euthanasia substituted for religiosity

| Dependant Variable | WTD | |
| --- | --- | --- |
|  | Coefficient | Standard Error |
| Third Level Education | -0.178 | (0.164) |
| Age Group (Base: 18-35) |  |  |
| 36-45 | 0.409 | (0.211) |
| 46-60 | 0.625** | (0.235) |
| 61+ | 1.467*** | (0.282) |
| Male | -0.357* | (0.166) |
| Married/Living as Married | -0.223 | (0.185) |
| Dependants Under 18 (Y/N) | 0.015 | (0.208) |
| VAS | 0.001 | (0.005) |
| Experienced a serious illness | -0.581** | (0.215) |
| Religiosity (Base: A few times a year or less) |  |  |
| Monthly | -0.136 | (0.219) |
| Weekly | -0.47* | (0.206) |
| Mobility (Base: No Problems) |  |  |
| Slight problems | 0.229 | (0.126) |
| Moderate problems | 0.373** | (0.121) |
| Severe problems | 0.531*** | (0.124) |
| Unable | 0.477*** | (0.114) |
| Self-care (Base: No Problems) |  |  |
| Slight problems | 0.287* | (0.145) |
| Moderate problems | 0.387** | (0.145) |
| Severe problems | 0.586*** | (0.149) |
| Unable | 0.49*** | (0.11) |
| Usual Activities (Base: No Problems) |  |  |
| Slight problems | 0.335* | (0.144) |
| Moderate problems | 0.535** | (0.163) |
| Severe problems | 0.489*** | (0.127) |
| Unable | 0.372*** | (0.098) |
| Paid/Discomfort (Base: No Problems) |  |  |
| Slight problems | 0.287* | (0.125) |
| Moderate problems | 0.207 | (0.129) |
| Severe problems | 0.68*** | (0.112) |
| Extreme problems | 0.94*** | (0.135) |
| Anxiety/Depression (Base: No Problems) |  |  |
| Slight problems | 0.242 | (0.148) |
| Moderate problems | 0.356* | (0.153) |
| Severe problems | 0.958*** | (0.15) |
| Extreme problems | 0.96*** | (0.116) |
| Constant | -2.522*** | (0.545) |
| Number of observations |  | 1600 |
| Number of clusters |  | 160 |

*p < 0.05, **p < 0.01, ***p < 0.001
